# Randomized controlled trials of digital health interventions for rheumatic disease management: a systematic review

**DOI:** 10.2471/BLT.24.292168

**Published:** 2024-12-11

**Authors:** Anindita Santosa, James Weiquan Li, Tze Chin Tan

**Affiliations:** a Aaria Rheumatology, 6 Napier Road 08-19, Gleneagles Medical Centre, Singapore 258499, Singapore.; b Department of Gastroenterology and Hepatology, Changi General Hospital, Singapore.; c Department of Rheumatology and Immunology, Singapore General Hospital, Singapore.

## Abstract

**Objective:**

To evaluate the adoption, effectiveness and cost-effectiveness of digital health interventions for rheumatic disease management.

**Methods:**

Between 25 May 2024 and 2 June 2024, we systematically searched PubMed®, Scopus, ClinicalTrials.gov, the Global Observatory for eHealth and the World Bank Open Knowledge Repository for randomized controlled trials (RCTs) evaluating digital health interventions for rheumatic disease management. We included studies published between 2000 and 2024 that reported on adoption rates and efficacy. Two reviewers independently screened the studies, extracted data and categorized the digital interventions according to the World Health Organization’s classification of digital health interventions.

**Findings:**

Of the 455 unique records identified, 21 RCTs met the inclusion criteria. Most digital health interventions (15 studies) focused on individual-centric interventions, such as targeted communication, personal health tracking, educational tools and wearable devices. Studies in high-income countries implemented advanced, integrated digital interventions combining individual-focused approaches with health worker interventions and data services using telemedicine platforms and decision support systems. In contrast, studies in low- and middle-income countries adapted accessible technologies such as mobile messaging and telephone-based education. Many telehealth, wearable technologies and educational interventions significantly improved disease control, patient adherence, knowledge and self-efficacy. Of the five studies assessing cost-effectiveness, all found digital interventions to be cost-effective.

**Conclusion:**

Digital health interventions show promise for managing rheumatic diseases. Tailoring these interventions to local infrastructure and emphasizing patient engagement are crucial for successful adoption. Future research should focus on standardizing evaluation methods, addressing digital divides and enhancing provider support and data services.

## Résumé

**Objectif:**

Évaluer l’adoption, l’efficacité et le rapport coût-efficacité des interventions de santé numérique pour la gestion des maladies rhumatismales.

**Méthodes:**

Entre le 25 mai 2024 et le 2 juin 2024, nous avons effectué une recherche systématique sur PubMed®, Scopus, ClinicalTrials.gov, l’Observatoire mondial de la santé en ligne (Global Observatory for eHealth) et le dépôt de connaissances ouvert de la Banque mondiale (World Bank Open Knowledge Repository) pour les essais contrôlés randomisés (ECR) évaluant les interventions de santé numérique pour la gestion des maladies rhumatismales. Nous avons inclus les études publiées entre 2000 et 2024 qui faisaient état des taux d’adoption et de l’efficacité. Deux évaluateurs indépendants ont examiné les études, extrait les données et classé les interventions numériques selon la classification des interventions de santé numérique de l’Organisation mondiale de la santé.

**Résultats:**

Sur les 455 dossiers uniques identifiés, 21 ECR répondaient aux critères d’inclusion. La plupart des interventions numériques en matière de santé (15 études) se sont concentrées sur des interventions centrées sur les individus, telles que la communication ciblée, le suivi personnel de la santé, les outils éducatifs et les dispositifs portables. Les études menées dans les pays à revenu élevé ont mis en œuvre des interventions numériques avancées et intégrées combinant des approches centrées sur les individus, avec des interventions de professionnels de la santé et des services de données ayant recours à des plateformes de télémédecine et des systèmes d’aide à la décision. En revanche, les études menées dans les pays à revenu faible ou intermédiaire ont adapté des technologies accessibles telles que la messagerie mobile et l’éducation par téléphone. De nombreuses interventions de télésanté, de technologies portables et d’éducation ont permis d’améliorer de manière significative le contrôle sanitaire, l’observance des patients, les connaissances et l’efficacité personnelle. Sur les cinq études évaluant le rapport coût-efficacité, toutes ont conclu que les interventions numériques étaient rentables.

**Conclusion:**

Les interventions de santé numérique sont prometteuses pour la gestion des maladies rhumatismales. Il est crucial d’adapter ces interventions aux infrastructures locales et de mettre l’accent sur la participation des patients pour que leur adoption soit une réussite. Les recherches futures doivent se concentrer sur la normalisation des méthodes d’évaluation, la réduction de la fracture numérique et l’amélioration du soutien aux prestataires et des services de données.

## Resumen

**Objetivo:**

Evaluar la adopción, eficacia y rentabilidad de las intervenciones de salud digital para el manejo de las enfermedades reumáticas.

**Métodos:**

Entre el 25 de mayo de 2024 y el 2 de junio de 2024, se realizaron búsquedas sistemáticas en PubMed®, Scopus, ClinicalTrials.gov, el Global Observatory for eHealth y el World Bank Open Knowledge Repository de ensayos controlados aleatorizados (ECA) que evaluaran intervenciones de salud digital para el manejo de enfermedades reumáticas. Se incluyeron estudios publicados entre 2000 y 2024 que informaban sobre las tasas de adopción y la eficacia. Dos revisores examinaron de forma independiente los estudios, extrajeron los datos y asignaron categorías a las intervenciones digitales según la clasificación de intervenciones de salud digital de la Organización Mundial de la Salud.

**Resultados:**

De los 455 registros únicos identificados, 21 ECA cumplieron los criterios de inclusión. La mayoría de las intervenciones de salud digital (15 estudios) se centraron en intervenciones centradas en el individuo, como la comunicación dirigida, el seguimiento personal de la salud, las herramientas educativas y los dispositivos portátiles. Los estudios realizados en países de ingresos altos aplicaron intervenciones digitales avanzadas e integradas que combinaban enfoques centrados en el individuo con intervenciones del personal sanitario y servicios de datos mediante plataformas de telemedicina y sistemas de apoyo a la toma de decisiones. En cambio, los estudios realizados en países de ingresos bajos y medios adaptaron tecnologías accesibles como la mensajería móvil y la educación por teléfono. Muchas intervenciones de telesalud, tecnologías portátiles y educación mejoraron de forma significativa el control de la enfermedad, la adherencia de los pacientes, los conocimientos y la autoeficacia. De los cinco estudios en los que se evaluó la rentabilidad, todos concluyeron que las intervenciones digitales eran rentables.

**Conclusión:**

Las intervenciones sanitarias digitales resultan prometedoras para el manejo de las enfermedades reumáticas. Adaptar estas intervenciones a la infraestructura local y fomentar la participación de los pacientes es crucial para que su adopción tenga éxito. Las investigaciones futuras deben centrarse en estandarizar los métodos de evaluación, abordar las diferencias digitales y mejorar el apoyo a los proveedores y los servicios de datos.

## ملخص

الغرض تقييم الاعتماد والفعالية والتكلفة المعقولة للتدخلات الصحية الرقمية لإدارة الأمراض الروماتيزمية.

الطريقة خلال الفترة بين 25 مايو/أيار 2024، و2 يونيو/حزيران 2024، قمنا بإجراء بحث بشكل منهجي في PubMed®‎، وScopus، وClinicalTrials.gov، ، والمرصد العالمي للصحة الإلكترونية (eHealth)، ومستودع المعرفة المفتوحة للبنك الدولي. وكان البحث عن التجارب العشوائية الخاضعة للتحكم (RCT) لتقييم التدخلات الصحية الرقمية لإدارة الأمراض الروماتيزمية. كما قمنا بتضمين الدراسات التي تم نشرها بين عامي 2000 و2024، والتي أوضحت معدلات التبني والفعالية. قام اثنان من المراجعين بشكل مستقل بفحص الدراسات، واستخراج البيانات، وتصنيف التدخلات الرقمية، وفقًا لتصنيف منظمة الصحة العالمية للتدخلات الصحية الرقمية.

النتائج من بين 455 سجلاً فريدًا تم تحديدها، استوفت 21 تجربة عشوائية معايير الاشتمال. ركزت أغلب التدخلات الصحية الرقمية (15 دراسة) على التدخلات التي تركز على الفرد، مثل الاتصالات المستهدفة، وتتبع الصحة الشخصية، والأدوات التعليمية، والأجهزة القابلة للارتداء. وقامت الدراسات في الدول ذات الدخل المرتفع بتنفيذ تدخلات رقمية متقدمة ومتكاملة، تجمع بين الأساليب التي تركز على الفرد، وتدخلات العاملين في القطاع الصحي، وخدمات البيانات باستخدام منصات العلاج الطبي عن بعد، وأنظمة دعم القرار. وعلى النقيض من ذلك، قامت الدراسات في الدول ذات الدخل المنخفض والدخل المتوسط بتكييف التقنيات المتاحة مثل رسائل الهاتف المحمول، والتعليم عبر الهاتف. وقد أدت العديد من الخدمات الصحية عن بعد، والتقنيات القابلة للارتداء، والتدخلات التعليمية، إلى التحسين بشكل كبير من السيطرة على المرض، والتزام المرضى، والمعرفة، والكفاءة الذاتية. ومن بين الدراسات الخمس التي قامت بتقييم فعالية التكلفة، فقد وجدت جميعها أن التدخلات الرقمية فعالة من حيث التكلفة.

الاستنتاج تظهر التدخلات الصحية الرقمية نتائج واعدة في إدارة الأمراض الروماتيزمية. إن تكييف هذه التدخلات مع البنية التحتية المحلية، والتأكيد على مشاركة المرضى، أمر بالغ الأهمية للتبني الناجح. وينبغي أن تركز الأبحاث المستقبلية على توحيد أساليب التقييم، ومعالجة الفجوات الرقمية، وتعزيز دعم مقدمي الخدمات، وخدمات البيانات.

## 摘要

**目的:**

旨在评估数字化卫生保健干预措施在风湿病管理中的采用情况、有效性和成本效益。

**方法:**

在 2024 年 5 月 25 日至 2024 年 6 月 2 日期间，我们系统地检索了美国国家医学图书馆的医学和生物医学文献数据库 (PubMed®)、斯高帕斯数据库 (Scopus)、ClinicalTrials.gov、【全球电子卫生保健观察站 (Global Observatory for eHealth)】和世界银行开放知识库 (World Bank Open Knowledge Repository)，以获取旨在评估用于风湿病管理的数字化卫生保健干预措施的随机对照试验 (RCT)。我们纳入了 2000 年至 2024 年间发表的报告了采用率和有效性的研究。两名评审员独立审查了这些研究、提取了相关数据，并根据世界卫生组织对数字化卫生保健干预措施的分类方法对这些数字化干预措施进行了归类。

**结果:**

在已确定的 455 份无重复记录中，有 21 项 RCT 符合纳入标准。大多数数字化卫生保健干预措施（15 项研究）是侧重于以个人为中心的干预措施，例如有针对性地沟通、个人健康状况追踪、教育工具和可穿戴设备。高收入国家开展的研究针对先进的综合性数字化干预措施，将以个人为中心的方法与卫生工作者依赖远程医疗平台和决策支持系统提供的干预措施和数据服务结合起来。相比之下，中低收入国家的研究则利用了手机短信和电话教育等无障碍技术。许多远程医疗服务、可穿戴技术和教育干预措施大幅提高了疾病控制能力、患者依从性、了解水平和自我效能。评估成本效益的五项研究结果均表明，数字化干预措施具有成本效益。

**结论:**

数字化卫生保健干预措施在管理风湿病方面展现出了良好的前景。根据当地基础设施调整相关干预措施并鼓励患者积极参与，这对于成功采用这些措施至关重要。未来的研究应侧重于规范评估方法、解决数字鸿沟问题以及增强供应商支持和数据服务能力。

## Резюме

**Цель:**

Оценить внедрение, эффективность и экономическую целесообразность медицинской помощи с применением цифровых технологий для лечения ревматических заболеваний.

**Методы:**

В период с 25 мая по 2 июня 2024 года был проведен систематический поиск в базах данных PubMed®, Scopus, ClinicalTrials.gov, Global Observatory for eHealth и World Bank Open Knowledge Repository на предмет рандомизированных контролируемых исследований (РКИ), оценивающих оказание медицинской помощи с применением цифровых технологий для лечения ревматических заболеваний. В список были включены исследования, опубликованные в период с 2000 по 2024 год, в которых сообщалось о показателях внедрения и эффективности. Два рецензента, независимо друг от друга, проверяли исследования, извлекали данные и классифицировали данные о медицинской помощи на основе цифровых технологий в соответствии с классификацией данных о корректировке здоровья на основе цифровых технологий, принятой Всемирной организацией здравоохранения.

**Результаты:**

Из 455 найденных уникальных записей 21 рандомизированное контролируемое исследование соответствовало критериям включения. Большинство случаев медицинской помощи с применением цифровых технологий (15 исследований) представляли собой индивидуальные вмешательства, такие как адресное взаимодействие, персональное отслеживание состояния здоровья, образовательные инструменты и носимые устройства. В исследованиях, проведенных в странах с высоким уровнем дохода, применялись передовые комплексные цифровые мероприятия, сочетающие индивидуальные подходы с вмешательством медицинских работников и предоставлением данных с помощью платформ для оказания телемедицинских услуг и систем поддержки принятия решений. В то же время в исследованиях, проведенных в странах с низким и средним уровнем дохода, использовались такие доступные технологии, как обмен мобильными сообщениями и обучение по телефону. Многие технологии телемедицины, носимые устройства и образовательные вмешательства значительно повысили эффективность контроля над заболеванием, приверженность пациента процессу лечения, знания и уверенность в собственных силах. По результатам пяти исследований, в которых оценивалась экономическая эффективность, все цифровые вмешательства были признаны экономически эффективными.

**Вывод:**

Мероприятия по оказанию медицинской помощи с применением цифровых технологий перспективны для лечения ревматических заболеваний. Адаптация этих вмешательств к местной инфраструктуре и акцент на вовлечении пациентов имеют решающее значение для успешного внедрения. Дальнейшие исследования должны быть направлены на стандартизацию методов оценки, устранение цифрового неравенства и расширение поддержки поставщиков и услуг по предоставлению данных.

## Introduction

Rheumatic diseases impose health burdens worldwide, with notable disparities between high- and low- and middle-income countries.^1^^,^^2^ In low- and middle-income countries, the burden of rheumatic diseases is exacerbated by constrained health-care resources and socioeconomic challenges, leading to higher mortality than in high-income countries.^3^ For example, fewer than 20 rheumatologists serve over 800 million people in the World Health Organization (WHO) African Region, resulting in inadequate diagnosis and treatment of these diseases.^4^^,^^5^

Addressing these disparities requires coordinated global efforts to ensure equitable access to effective treatments for rheumatic diseases worldwide. WHO and the International League of Associations for Rheumatology have initiated programmes to document and address the prevalence of rheumatic diseases in low- and middle-income countries, highlighting the need for better education for patients, their family and other stakeholders, and improved health-care infrastructure.^6^^,^^7^

Digital health interventions have shown considerable promise for patients with rheumatic disease, by improving health outcomes, increasing access to care and reducing health-care costs.^8^^–^^11^ These interventions have emerged as valuable tools for improving rheumatic disease management by enhancing patient engagement, monitoring and communication with health workers.^12^^,^^13^ The expectation is that the adoption of these technologies will transform the delivery of rheumatic care in various health-care settings.^14^

In high-income countries, digital health interventions have been extensively studied and found to be cost-effective for managing chronic diseases and promoting behavioural changes, such as smoking cessation and obesity management.^15^^,^^16^ These interventions often use mobile health applications, text messaging and online platforms to deliver health-care services efficiently and at low costs.^16^ For example, in the Republic of Korea, digital health interventions have been recommended for obesity management, showing potential for scalable and cost-effective treatment.^17^ In contrast, the use of digital health interventions in low- and middle-income countries is less widespread and under-researched, particularly in primary health-care settings. A 2023 scoping review highlighted that only 14 of the 28 digital health intervention categories classified by WHO were used in low- and middle-income countries, indicating a considerable gap in innovation and application.^15^

Despite the growing interest in digital health interventions for managing rheumatic diseases, comprehensive evidence is needed to assess their adoption and effectiveness across different health-care settings.^12^ Such evidence is also important for informing the development of implementation strategies and optimizing these interventions.^11^^,^^18^

Here, we present the results of a systematic review assessing the current state of adoption and effectiveness of digital health interventions for managing rheumatic diseases across different settings.

## Methods

We performed a systematic review to evaluate the effectiveness, implementation patterns and cost-effectiveness of digital health interventions for autoimmune rheumatic disease management, using the Preferred Reporting Items for Systematic Reviews and Meta-Analysis guidelines. We registered the review with PROSPERO (CRD42024547195). 

### Search

We integrated relevant keywords and medical subject headings pertaining to digital health interventions, autoimmune rheumatic conditions and national income classification in our search protocol (Box 1). The search criteria included digital health interventions; rheumatic disorders; country income categories; and terms related to their implementation, efficacy, adoption and administration. We searched PubMed®, Scopus and ClinicalTrials.gov, as well as grey literature repositories, such as the Global Observatory for eHealth and the World Bank Open Knowledge Repository between 25 May 2024 and 2 June 2024 for articles published in any language between 2000 and 2024.

Box 1 **Search strategy for systematic review of digital health interventions in the management and implementation of rheumatic diseases**

**PubMed®**

(“Digital health interventions*” OR “Telemedicine” OR “Chatbots*” OR “Wearable technologies*” OR “AI-powered predictive tools*” OR “Artificial intelligence”)

AND

(“Rheumatic diseases*” OR “Rheumatoid arthritis” OR “Lupus” OR “Ankylosing spondylitis” OR “Psoriatic arthritis”)

AND

(“Adoption” OR “Effectiveness” OR “Efficacy” OR “Implementation” OR “Management”)

**ClinicalTrials.gov**

("Rheumatic" OR "Rheumatoid arthritis" OR "Systemic lupus erythematosus" OR "SLE" OR "Ankylosing spondylitis" OR "Psoriatic arthritis" OR "Autoimmune arthritis" OR "Inflammatory arthritis" OR "Spondyloarthritis" ) | Other terms: ( "Digital health" OR "Digital health intervention" OR "eHealth" OR "mHealth" OR "Telemedicine" OR "Telehealth" OR "Remote monitoring" OR "Chatbot" OR "Virtual assistant" OR "Wearable" OR "Mobile app" OR "Digital therapeutic" OR "Artificial intelligence" OR "Machine learning" OR "AI" ) | Digital health

**Scopus**

TITLE-ABS-KEY (( "Digital health" OR "Digital health intervention*" OR "eHealth" OR "mHealth" OR "Telemedicine" OR "Telehealth" OR "Remote monitoring" OR "Chatbot*" OR "Virtual assistant*" OR "Wearable" OR "Mobile app*" OR "Digital therapeutic" OR "Artificial intelligence" OR "Machine learning" OR "AI" ) AND ( "Rheumat" OR "Rheumatoid arthritis" OR "Systemic lupus erythematosus" OR "SLE" OR "Ankylosing spondylitis" OR "Psoriatic arthritis" OR "Autoimmune arthritis" OR "Inflammatory arthritis" OR "Spondyloarthritis" ) AND( "Adopt" OR "Accept" OR "Effect" OR "Efficacy" OR "Implementation" OR "Implement" OR "Manag*" OR "Outcome" OR "Impact" OR "Feasibility" OR "Usability" OR "Adherence" ))"

### Eligibility criteria

We included studies if they (i) involved individuals living with autoimmune rheumatic conditions; (ii) evaluated digital health interventions; (iii) documented adoption levels or efficacy in managing rheumatic disorders; and (iv) were randomized controlled trials (RCT) detailing the effectiveness of digital health interventions. Conversely, we excluded studies if they (i) did not primarily address rheumatic diseases; (ii) lacked digital health intervention components; (iii) failed to provide clear information on country-specific implementation or economic classification; (iv) were categorized as systematic reviews, meta-analyses or academic theses; and (v) merely described digital health interventions without assessing their implementation or efficacy.

### Selection and data extraction

Using the eligibility criteria, two reviewers independently screened the titles and abstracts of all identified records. Disagreements were resolved through discussion or by involving a third reviewer. Two reviewers independently assessed the full texts of potentially eligible studies, with conflicts resolved using the same approach. Two reviewers independently extracted data using a standardized form that included the study characteristics, participant characteristics, interventions and outcomes.

### Classification of interventions

We classified identified digital health interventions according to WHO *Classification of digital interventions, services and applications in health* (second edition).^19^ We mapped each intervention into one or more of the four domains: individuals; health workers; health system managers; and data services. In each article, we identified primary user groups and predominant functions for classification purposes, with crosscutting interventions categorized based on their principal operational focus.

### Risk of bias

Two reviewers independently assessed the risk of bias using RoB 2, the Cochrane risk-of-bias tool for RCTs.^20^ They evaluated five domains: randomization process; deviations from intended interventions; missing outcome data; measurement of outcomes; and selection of reported results. We rated each domain as either low risk, some concerns or high risk.

### Digital health framework 

Drawing upon insights from the WHO Digital health implementation framework,^21^ we used the findings from the review to conceptualize a framework delineating dimensions of digital health interventions in rheumatology.

## Results

### Study characteristics

**Fig. 1:**
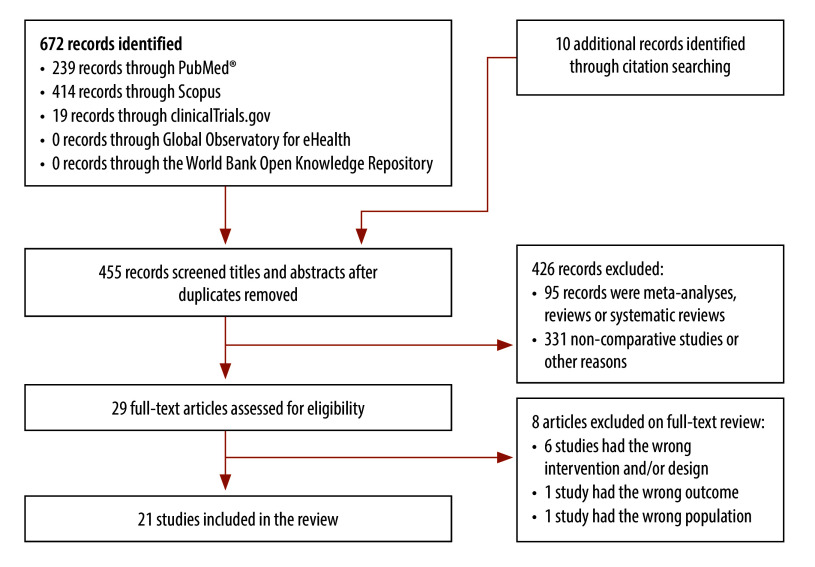
Flowchart illustrating the selection of studies included in the review on digital health interventions for rheumatic disease management

Our search yielded 672 records. After removing duplicates, we screened 455 unique records and obtained 29 records for full-text review (Fig. 1). Of these, 21 RCTs met our eligibility criteria.^22^^–^^42^ The included studies were published between 2013 and 2023, and most studies (13)^24^^–^^28^^,^^34^^–^^40^^,^^42^ had been conducted in high-income countries. The sample size of the included studies ranged from 30 to 320, and follow-up time ranged from 1 to 12 months (Table 1). 

**Table 1:** Characteristics of included studies on digital health interventions in rheumatology, categorized by WHO digital health intervention classifications^19^

Study, income category	Country	Sample size total (intervention/control)	Disease	Digital health intervention		Follow-up, months
				Type	WHO classification	
**High-income country**						
Rimmer et al., 2013^38^	USA	102	Various disabilities	Telehealth weight management	Targeted communication to individuals (1.1) Person-to-person communication (1.3) Person-centred longitudinal records (2.2)	NR
Salaffi et al., 2016^37^	Italy	41	Rheumatoid arthritis	Telemonitoring	Identification and registration of health services (2.1) Person-centred longitudinal records (2.2) Data management (4.1)	12
Gosse et al., 2018^34^	France	320	Rheumatoid arthritis	eHealth platform	Targeted communication to individuals (1.1) Person-to-person communication (1.3)	12
Thurah et al., 2018^35^	Denmark	294	Rheumatoid arthritis	Patient-reported outcomes by telehealth	Identification and registration of health services (2.1) Person-centred longitudinal records (2.2)	12
Taylor-Gjevre et al., 2018^36^	Canada	85	Rheumatoid arthritis	Videoconferencing for follow-up	Person-centred longitudinal records (2.2)	9
Boedecker et al., 2020^40^	Germany	30	Systemic lupus erythematosus	Internet-based 12-week exercise programme	Targeted communication to individuals (1.1) Person-to-person communication (1.3)	3
Khan et al., 2020^42^	USA	50	Systemic lupus erythematosus	Digital app and telehealth coaching	Targeted communication to individuals (1.1) Person-to-person communication (1.3) Person-centred longitudinal records (2.2)	NR
Pers et al., 2021^28^	France	89	Rheumatoid arthritis	App for connected monitoring versus conventional monitoring	Person-to-person communication (1.3) Identification and registration of health services (2.1) Data management (4.1)	6
Bernard et al., 2022^27^	France	89	Rheumatoid arthritis	App for connected monitoring	Person-to-person communication (1.3) Identification and registration of health services (2.1) Data management (4.1)	6
Lopez-Olivo et al., 2022^26^	USA	210	Rheumatoid arthritis	Facebook peer group and educational website	Targeted communication to individuals (1.1) Untargeted communication to individuals (1.2)	6
Pouls et al., 2022^25^	Kingdom of the Netherlands	221	Rheumatoid arthritis	Gaming and disease-modifying antirheumatic therapy	Targeted communication to individuals (1.1) Person-to-person communication (1.3)	NR
Rodríguez Sánchez-Laulhé, et al., 2022^24^	Spain	36	Rheumatoid arthritis	App for hand exercise and self-management	Targeted communication to individuals (1.1) Person-to-person communication (1.3)	6
Skovsgaard et al., 2023^39^	Denmark	294	Rheumatoid arthritis	Follow-up using telehealth. Patient reporting outcomes to a rheumatologist or a nurse	Identification and registration of health services (2.1) Person-centred longitudinal records (2.2) Data management (4.1)	12
**Low- and middle-income country**						
Zhao et al., 2019^33^	China	92	Rheumatoid arthritis	Telephone-based health education	Targeted communication to individuals (1.1)	6
Song et al., 2020^41^	China	92	Rheumatoid arthritis	Telephone-based health education	Targeted communication to individuals (1.1)	6
Adly et al., 2021^30^	Egypt	60	Rheumatoid arthritis	Laser acupuncture and telerehabilitation	Identification and registration of health services (2.1) Person-centred longitudinal records (2.2)	NR
Song et al., 2021^32^	China	118	Ankylosing spondylitis	WeChat-based education	Targeted communication to individuals (1.1)	3
Adly et al., 2022^29^	Egypt	60	Rheumatoid arthritis	Laser acupuncture and telerehabilitation	Identification and registration of health services (2.1) Person-centred longitudinal records (2.2)	NR
Song et al., 2022^31^	China	118	Ankylosing spondylitis	Health education via WeChat app	Targeted communication to individuals (1.1) Person-to-person communication (1.3)	3
Sunthornsup et al., 2022^23^	Thailand	100	Juvenile idiopathic arthritis	Brochure versus video education	Targeted communication to individuals (1.1)	1
Wang et al., 2022^22^	China	55	Ankylosing spondylitis	Wearable-assisted home exercise	Targeted communication to individuals (1.1) Person-to-person communication (1.3)	4

App: application; NR: not reported; WHO: World Health Organization.

**Fig. 2:**
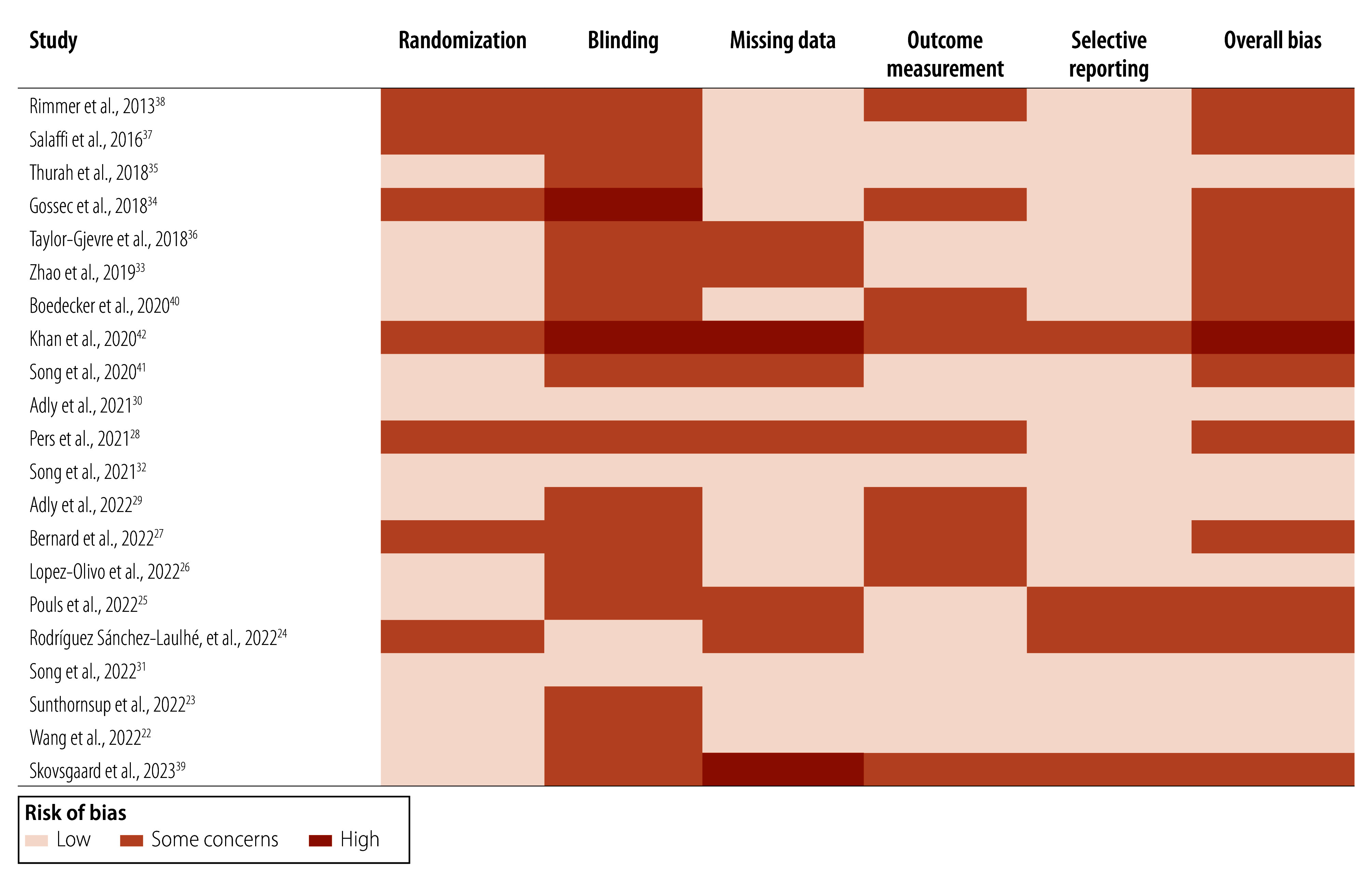
Risk of bias in the studies included in the systematic review on digital health interventions for rheumatic disease management

Notes: we used RoB 2, which is Version 2 of the Cochrane risk-of-bias tool for randomized trials.20 Low risk indicates that the study adequately addressed the domain with minimal potential for bias. Some concerns indicates that there is a possibility of bias due to limited information or methodological shortcomings. High risk indicates a significant potential for bias affecting the study's validity.

The quality assessment revealed robust randomization processes in the studies, although methodological challenges were noted, particularly in blinding procedures and outcome data completeness (Fig. 2).

### Digital health interventions 

Among the digital interventions reviewed, nine studies evaluated application (app)-based interventions,^24^^,^^25^^,^^27^^–^^32^^,^^42^ followed by eight studies focusing on telehealth interventions,^23^^,^^33^^,^^35^^–^^39^^,^^41^ three studies evaluated platform-based interventions^26^^,^^34^^,^^40^ and one study assessed wearable technology.^22^

When aligning the identified digital health interventions in the studies with the WHO classification, we noticed distinct implementation categories (Table 1). Most studies focused on individual-focused interventions, such as targeted communication and personal health tracking, leveraging mobile apps, educational tools and wearable devices to enhance patient engagement and self-management. High-income countries predominantly implemented advanced, integrated digital health interventions combining individual-focused approaches with health worker interventions and data services, using robust telemedicine platforms and decision support systems. In contrast, low- and middle-income countries effectively adapted accessible technologies for individual-focused interventions, such as mobile messaging and telephone-based education, to overcome resource constraints. 

### Outcomes

Various interventions showed successful outcomes (Table 2). For example, a wearable technology intervention achieved 84.2% median adherence (interquartile range: 48.7–97.9) to ankylosing spondylitis management protocol.^22^

**Table 2:** Clinical outcomes, cost-effectiveness, and safety of digital health interventions in rheumatology

Study	Disease	Intervention	Key outcomes	Adoption and satisfaction	Cost–effectiveness	Adverse events
**High-income country**						
Rimmer et al., 2013^38^	Various Disabilities	Telehealth weight management	Decreased weight, improved diet and fewer barriers	Moderate; high engagement	Cost-effective telehealth	None
Salaffi et al., 2016^37^	Rheumatoid arthritis	Telemonitoring versus conventional	Increased remission and shorter time to remission	High satisfaction	NA	None
Gossec et al., 2018^34^	Rheumatoid arthritis	eHealth platform	Increased patient–physician interactions and patient satisfaction	High; limited reuse	NA	None
Thurah et al., 2018^35^	Rheumatoid arthritis	Patient reporting outcomes by telehealth versus conventional	Disease Activity Score-28 non-inferior, decreased hospital visits	High; equal to conventional	Likely cost-effective	None
Taylor-Gjevre et al., 2018^36^	Rheumatoid arthritis	Videoconferencing	No change in disease control and increased dropout	Moderate; high satisfaction	Cost-effective rural focus	None
Boedecker et al., 2020^40^	Systemic lupus erythematosus	Internet-based exercise programme	Increased maximal O_2_ consumption, decreased fatigue and depression levels	Positive adherence and satisfaction	NA	None
Khan et al., 2020^42^	Systemic lupus erythematosus	Digital app and telehealth	Improved health-related quality of life (less fatigue and pain), high adherence	High; symptom tracking effective	Potential savings	None
Pers et al., 2021^28^	Rheumatoid arthritis	Connected versus conventional monitoring	Fewer visits and increased quality of life	High; visits down significantly	Cost-effective	One unrelated event
Bernard et al., 2022^27^	Rheumatoid arthritis	Connected versus conventional monitoring	Lower cost, improved quality of life and quality-adjusted life years	High; fewer visits	Highly cost-effective	None
Lopez-Olivo et al., 2022^26^	Rheumatoid arthritis	Facebook and website	Increased peer support satisfaction	Moderate; high satisfaction	Potentially cost-effective	None
Pouls et al., 2022^25^	Rheumatoid arthritis	Gaming and disease-modifying antirheumatic therapy	High engagement, no change in adherence	High; median play 9.7h	Low-cost ehealth	None
Rodríguez Sánchez-Laulhé, et al., 2022^24^	Rheumatoid arthritis	App for hand exercise and self-management	Improved Michigan Hand Outcomes scores and less pain	High; regular use	Fewer in-person care visits	None
Skovsgaard et al., 2023^39^	Rheumatoid arthritis	Patient reporting outcomes to a rheumatologist or a nurse using telehealth	Lower costs and no change in disease control	High; decreased visits	Savings with using either a telehealth rheumatologist or nurse compared to conventional outpatient follow-up	None
**Low- and middle-income country**						
Zhao et al., 2019^33^	Rheumatoid arthritis	Telephone-based health education	Increased self-efficacy, no change in Disease Activity Score-28 and Health Assessment Questionnaire scores	High; positive feedback	Cost-effective remote support	None
Song et al., 2020^41^	Rheumatoid arthritis	Telephone-based health education	Increased medication adherence	High; positive feedback	Potentially cost-effective	None
Adly et al., 2021^30^	Rheumatoid arthritis	Laser acupuncture and telerehabilitation	Decreased levels of IL-6, MDA and CRP; improved rheumatoid arthritis quality of life and increased level of ATP	High; preferred to in-person	Remote management. Reduced costs	None
Song et al., 2021^32^	Ankylosing spondylitis	WeChat education	Improved quality of life and decreased depression	High; preferred format	Cost-effective WeChat	None
Adly et al., 2022^29^	Rheumatoid arthritis	Laser acupuncture and telerehabilitation	Consistent outcomes across a range of health assessments. Improvements in measures such as rheumatoid arthritis quality of life, and key biomarkers including CRP and IL-6	High; high	Cost-effective	None
Song et al., 2022^31^	Ankylosing spondylitis	Health education via WeChat app	Increased knowledge, self-efficacy and exercise	High; positive feedback	Cost-effective (mobile)	None
Sunthornsup et al., 2022^23^	Juvenile idiopathic arthritis	Video versus brochure	Increased immediate knowledge	High; more effective in participants with limited education	Cost-effective education	None
Wang et al., 2022^22^	Ankylosing spondylitis	Wearable-based exercise	Improvement in ankylosing spondylitis disease activity score and Bath ankylosing spondylitis disease activity index and improved quality of life	High (84% adherence); positive feedback	Cost-effective; broad reach	Minor

ATP: adenosine triphosphate; CRP: C-reactive protein; IL-6: interleukin-6; MDA: malondialdehyde; NA: not applicable; O_2_: oxygen.

Notes: key outcomes cover health-related benefits. Adoption and satisfaction include patient and provider engagement and experience. Cost–effectiveness indicates economic feasibility or savings.

Several studies reported on outcomes from educational interventions. In Thailand, video-based education improved juvenile idiopathic arthritis knowledge more than brochure-based education, with score increases of 4.44 versus 3.74 points, respectively.^23^ In China, app-based education through WeChat improved disease knowledge and quality of life,^31^^,^^32^ whereas telephone education enhanced self-efficacy.^33^ Using a social networking group for rheumatoid arthritis education did not result in higher knowledge than the control group, but participants in the network group had higher self-efficacy (*P*-value: 0.02).^26^

Medication adherence was a reported outcome in some studies. One study reported improved medication adherence through telehealth educational interventions, with significantly higher adherence at 12 weeks (72.87% versus 63.79%; *P*-value: 0.014).^41^ The implementation of a gaming application was associated with a non-significant 9% increase in medical adherence relative to the control group.^25^

In Egypt, patients receiving an acupoint detector, allowing them to receive laser therapy interventions at home, and installing an app to communicate with health workers, had significantly improved quality of life (*P*-value < 0.05).^29^^,^^30^

A French self-assessment and self-monitoring platform improved patient-perceived patient–physician interaction.^34^ In two studies, telehealth consultations demonstrated non-inferiority compared to conventional outpatient follow-up.^35^^,^^36^

One of the earliest interventions reported that people living with disabilities who participated in a weight management programme incorporating a web-based remote coaching tool and regular coaching telephone calls lost body weight.^38^

#### Disease-specific outcomes

A Danish study showed that patients reporting outcomes through telephone-based follow-ups had similar disease control compared to those receiving conventional care. The telehealth group showed a mean change in disease activity score of −0.0 versus −0.19 in the conventional group at 12 months. The authors concluded that the level of disease control was similar between patients managed by rheumatologists and those managed by rheumatology nurses.^35^ This finding was substantiated by a study reporting superior outcomes with telemonitoring compared to conventional outpatient follow-ups. The telemonitoring group achieved higher remission levels (38.1% versus 25.0%; *P*-value < 0.01) and shorter time to remission (median 20 versus 36 weeks; *P*-value < 0.001).^37^ Another study demonstrated that monitoring through smartphones significantly reduced the total number of hospital visits between baseline and six months (0.42 versus 1.93; *P*-value < 0.05) while maintaining disease control.^28^ A self-management app-based programme improved hand function, with a significant time × group effect observed for the total Michigan hand questionnaire score (*P*-value < 0.001) and subscales like hand function, work performance, pain and satisfaction (all *P*-value < 0.05). Mean differences in total scores were 16.86 points at 3 months and 17.21 points at 6 months.^24^

A wearable technology-assisted home exercise programme has showed significant improvements in the ankylosing spondylitis disease activity score (−0.2; 95% confidence interval: −0.4 to −0.02) Additionally, at 16 weeks, notable improvements were observed in secondary outcomes, such as pain levels, fatigue, spinal pain and mobility, and morning stiffness.^22^

In patients with systemic lupus erythematosus, a trial showed that internet-based individualized exercise programmes were safe, with no serious adverse events reported, and 25 of the 30 participants completing the study.^40^ Another randomized controlled pilot study evaluated the effectiveness of a digital therapeutic intervention combined with usual care, compared to usual care alone in improving quality of life for with systemic lupus erythematosus patients. The intervention group showed significantly greater improvements in 9 out of 11 health-related quality of life domains. Key outcomes included a 34% improvement in fatigue scores compared to 1% in the control group (*P*-value < 0.001), a 25% versus 0% improvement in pain interference (*P*-value: 0.02), and gains in emotional health, planning and reduced burden to others.^42^ These findings indicate the potential of digital therapeutic approaches to enhance health-related quality of life in systemic lupus erythematosus patients. 

#### Cost-effectiveness

Several studies conducted in high-income countries have evaluated the cost-effectiveness of digital health interventions versus conventional care for patients with rheumatic diseases. A Danish study using a telehealth intervention for patient-reported outcomes found that reporting to a rheumatologist was less costly than conventional follow-up, whereas reporting to a nurse had similar costs as conventional care.^39^ Two studies conducted in France found connected monitoring for rheumatic arthritis to be highly cost-effective, in contrast with the lower cost-effectiveness of conventional monitoring.^27^^,^^28^ In Canada, one study found video conferencing for rheumatic arthritis management to be more cost-effective than in-person care.^36^ Authors of an Italian study suggested that intensive telemonitoring was more cost-effective than conventional management, though exact figures were not provided.^37^

### Country comparison 

Studies conducted in both high-income and low- and middle-income countries demonstrated improvements in various outcome measures, including disease activity scores,^22^^,^^31^^,^^39^ physical function,^24^^,^^29^^,^^30^ quality of life,^32^^,^^42^ pain^24^^,^^42^ and fatigue.^42^ However, studies from high-income countries also evaluated additional outcomes, such as cost-effectiveness,^39^ patient satisfaction,^28^^,^^35^^,^^36^ health-care resource utilization^28^ and patient-physician interactions.^34^ There was no difference in follow-up time between studies conducted in high-income versus low-and middle-income countries (Table 1).

The type of digital intervention and implementation approach varied between high-income and low- and middle-income countries. Studies from high-income countries predominantly used advanced telehealth systems, mobile applications for disease monitoring, and eHealth platforms, often integrating features such as person-to-person communication, data management, and patient-centred longitudinal records.^27^^,^^35^^,^^37^Conversely, studies from low- and middle-income countries frequently focused on simpler digital tools, such as health education through messaging apps or telephone-based interventions.^31^^,^^33^ The success of digital health interventions appears to be influenced by contextual factors, including country income levels, digital literacy and the availability of supporting health-care infrastructure.^23^^,^^29^^,^^30^

In both high-income and low- and middle-income countries, digital health interventions were generally well received by participants. Several studies from high-income countries reported high levels of adoption and engagement,^24^^,^^25^ although some noted mixed engagement or lacked detailed information on adoption levels.^26^ Studies conducted in low- and middle-income countries also typically reported high levels of engagement and participation, particularly for educational interventions delivered via mobile apps or telephone.^22^^,^^23^^,^^31^^,^^33^^,^^41^

### Digital health framework

**Fig. 3:**
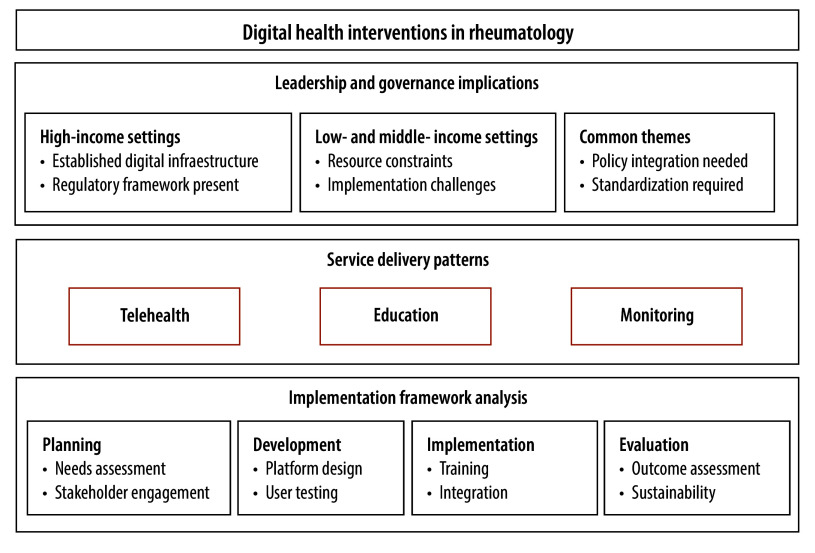
Framework for digital health implementation in rheumatology care

Notes: we adapted the framework from World Health Organization digital health implementation guidance.^21^ Intervention types presented in service delivery patterns box are types identified in this review.

Our framework, shown in Fig. 3, delineates three critical dimensions of digital health interventions in rheumatology. The first dimension, leadership and governance, shows that high-income countries benefit from established digital infrastructure and regulatory frameworks. In contrast, low- and middle-income countries face substantial resource constraints, underscoring the need for context-specific policy development. For the second dimension, we have identified telehealth, education, and monitoring digital health interventions as the predominant modalities for service delivery in rheumatology, with the type of intervention depending on health-care priorities and resource availability in the country. The third dimension focuses on the implementation process, which follows a structured four-stage approach: planning, development, implementation and evaluation. Low- and middle-income countries often require more needs assessments during planning due to limited infrastructure, while high-income countries typically face more challenges in integrating complex systems during implementation.

## Discussion

Through a systematic review, we analysed RCTs evaluating digital health interventions for managing rheumatic diseases across various economic settings, highlighting both the advancements and challenges in digital rheumatology care. The adoption of digital health interventions shows promising outcomes in both high-income countries and low- and middle-income countries, with variations in their implementation and outcomes. High-quality RCTs from high-income countries demonstrated significant clinical benefits.^27^^,^^39^ In low- and middle-income countries, innovative adaptations of accessible technologies achieved health improvements,^22^ demonstrating the potential for cost-effective digital solutions in resource-constrained settings.

We observed a difference in technological sophistication and implementation approaches between high-income countries and low- and middle-income countries. While studies from high-income countries evaluated advanced digital platforms,^27^^,^^39^ implementation in low- and middle-income countries focused on more accessible interventions, such as telephone-based health education^33^ and social media-based programmes.^32^ This disparity extends beyond technological differences to include variations in the health system infrastructure, user engagement patterns and implementation strategies. These differences show the importance of tailoring digital health interventions to the specific infrastructure and needs of each setting. While emphasizing patient engagement across all contexts, there are opportunities to improve provider support and data services, particularly in resource-constrained contexts. Therefore, adapting digital solutions to local contexts and available resources is a critical factor for intervention efficacy, particularly in resource-constrained settings. 

Cost–effectiveness analyses from high-income countries demonstrated promising economic outcomes. Two studies showed comparable clinical outcomes and cost–effectiveness of telehealth interventions, although methodological approaches varied considerably across health systems and implementation contexts.^35^^,^^36^

Notably, despite the overall promising results of the digital health interventions, our analysis revealed gaps in methodological rigour. Only a limited number of self-management applications have been evaluated through RCTs, with many studies limited to pilot trials or feasibility assessments.^43^^,^^44^ This limitation is particularly evident in newer mobile applications and patient-facing tools with scarce robust effectiveness data. Heterogeneity in outcome measures and implementation strategies further complicates cross-study comparisons, as illustrated by contrasting findings between studies.^26^ Methods for safety monitoring varied across studies, warranting careful consideration in clinical implementation. Although many trials reported no adverse events,^23^^,^^27^ others have documented important safety concerns, including misdiagnoses in telemedicine consultations^45^ and increased pain in remote monitoring programmes.^46^

This review has some limitations. Considerable heterogeneity in study designs, intervention types, outcomes assessed and follow-up durations complicates comparisons of studies and result synthesis. Methodological concerns, including inadequate reporting of randomization and blinding, particularly in studies from low- and middle-income countries, were prevalent. We could only assess cost–effectiveness data from high-income countries, limiting the applicability of the findings to resource-constrained settings. Short follow-up periods restricted the ability to evaluate long-term outcomes and the sustainability of the interventions. Inconsistent adoption metrics and different outcome measures further hindered cross-study comparisons. Additionally, the review’s focus on RCTs excluded valuable insights from nonrandomized or observational studies, which could provide a comprehensive understanding of real-world challenges and facilitators. Addressing these limitations in future studies will enhance the generalizability, scalability and equity of digital health interventions for managing rheumatic diseases.

In conclusion, various digital health interventions for rheumatic disease management have been evaluated across economic settings. While evidence supports their effectiveness, decision-makers should carefully consider the methodological rigour of the RCTs, safety and context-specific strategies when integrating digital interventions into clinical practice.

The digital divide between high-income countries and low- and middle-income countries presents challenges to achieving equitable access to digital health solutions, including disparities in financial resources and technical competencies. However, global collaboration in digital health offers opportunities to harness the complementary strengths of different countries and foster international partnerships. For example, such collaborations could enable data sharing, facilitate knowledge sharing, and support the development of advanced algorithms, software and applications that address diverse health-care challenges across varying contexts. Organizations such as WHO, governmental bodies and private stakeholders could play a pivotal role in addressing these disparities to promote equitable access to digital health solutions.
